# Serotonin Transporter Gene Polymorphisms and Maternal Overprotection Regulate Adult Social Expectations on Close Relationships

**DOI:** 10.3390/brainsci11091123

**Published:** 2021-08-25

**Authors:** Andrea Bonassi, Ilaria Cataldo, Giulio Gabrieli, Bruno Lepri, Gianluca Esposito

**Affiliations:** 1 Department of Psychology and Cognitive Science, University of Trento, 38068 Rovereto, Italy; andrea.bonassi@unitn.it (A.B.); ilaria.cataldo@unitn.it (I.C.); 2 Mobile and Social Computing Lab, Bruno Kessler Foundation, 38123 Trento, Italy; lepri@fbk.eu; 3 Psychology Program, School of Social Sciences, Nanyang Technological University, Singapore 639818, Singapore; giulio001@e.ntu.edu.sg; 4 Lee Kong Chian School of Medicine, Nanyang Technological University, Singapore 636921, Singapore

**Keywords:** gene*environment, serotonin transporter gene, 5HTTLPR, attachment, parent-infant interaction, parental bonding, maternal overprotection, close relationship, anxiety, avoidance

## Abstract

Humans are evolutionary-driven to adult mating and conceive social expectations on the quality of their affiliations. The genetic susceptibility to adverse environments in critical periods can alter close relationships. The current research investigates how the promoter region of the Serotonin Transporter Gene (5-HTTLPR) and perceived caregiving behavior in childhood could influence the social expectations on close adult relationships. For this purpose, 5-HTTLPR data was collected from the buccal mucosa of 65 Italian individuals (33 males). The participants filled (a) the Parental Bonding Instrument (PBI) to provide the levels of care and overprotection from mother and father, and (b) the Experience in Close Relationships-Revised (ECR-R) to report the social expectations on the intimate relationship assessed in terms of anxiety and avoidance from the partner. An interaction effect between 5-HTTLPR and PBI dimensions on the ECR-R scores was hypothesized. Results confirmed that the interplay between the genetic groups and history of maternal overprotection predicted avoidance experienced in romantic relationships in adulthood. Moreover, both adult anxiety and avoidance felt in an intimate relationship were found to covary as a function of maternal overprotection. The present work proposes further evidence of the genetic and parental mechanisms regulating social expectations involved in close relationships.

## 1. Introduction

In the last two decades, a large number of studies explored the complex set of mechanisms that shape social development in humans [1,2]. Among the primary mechanisms, genetic predispositions and biological characteristics, as well as psychological and environmental factors play a fundamental role in the development of social bonds from childhood to adulthood [3,4].

### 1.1. Parental Bonding and Adult Attachment across Human Development

Across the developmental stages, people are involved in relationships with significant social actors. From birth, the baby can rely on caregivers’ ability to address the physiological needs and adjust the psychological demands. The parental bond finds its origin in the evolutionary co-regulation of physiological and endocrine signals among mother and baby [5,6]. The same requests for care, protection, and support in the first years of life arise from the urgency to promote social ties. Parental education drives the flourishing of social competencies starting from infancy, where family represents the first social environment where the infant freely plays and socially interacts with parents [7,8]. There is robust evidence in many domains of psychology that recognizes the early interactions with the primary caregivers have a considerable impact on child development, in the mechanisms that drive social interactions across lifespan [7,9,10,11], and psychological well-being [12,13]. The quality of early relationships with parents as prominent social agents fosters the development of social abilities. Positive caregiving practices enhance early attachment with parents and lead to improved self-esteem, prosocial behavior, and a higher propensity to undertake social relationships throughout human development [9,13,14]. Moreover, previous studies support the evidence that optimal parenting qualities contribute to developing more adaptive skills related to emotion regulation and processing [15,16]. According to the literature, ideal parenting practices can be described in terms of high levels of care and low levels of over-protection. While parental care supports a child’s self-expression and exploration of new social situations, overprotection can be perceived as restrictive or excessive vigilance, reducing the possibility of building adequate emotion regulation processes with further consequences on social interactions [7,9,13,17]. As a consequence, less adaptive parenting practices can be the early core of later anxiety and avoidance experienced in social relationships [18,19]. A recent investigation on two different cultural contexts found that maternal overprotection perceived during childhood was associated with increased anxiety in close adult relationships in a Western-representative country [20], while greater reported levels of maternal care were correlated to lower experienced avoidance [20]. A previous study by Riskind and colleagues highlights that increased paternal and low maternal overprotection levels were predictive of a cognitive vulnerability to preoccupation and anxiety [21]. Another research conducted by Sun and colleagues [22] found different patterns in the participants displaying more optimal adult attachment, in males reporting greater parental care and lower overprotection and in females recalling higher levels of paternal care only.

### 1.2. Genotype 5HTTLPR and Attachment: Effects on Human Development

If on one side the early environment represents one of the major elements occurring in the development of social behavior, it is crucial to consider that genetic factors play a role in conferring different levels of sensitivity towards the environment. Specifically, the modulation of functions related to social behavior implies the genetic regulation of neurotransmitters proved to be implicated in social behavior. Concerning this, the serotonin transporter-linked polymorphic region (5-HTTLPR) is a polymorphic region within the SLC6A4 gene and can present two allelic variants, namely long (L) and short (S) alleles [23,24]. This genotype is involved in the mechanisms that regulate serotonin levels. The S-allele variation has been found to be associated with decreased 5-HTT expression, hence with lower serotonin re-uptake compared to the L-allele variation [23,25]. Individuals carrying the S-allele variation show greater brain activation in response to stimuli with emotional valence [26], and increased stress sensitivity, which can lead to negative outcomes, such as anxiety and sad mood [27], especially when unpleasant stimuli are presented [24,26,28]. Conversely, the L-variation has been linked to more pronounced social adaptiveness [29], such as reduced intergroup bias [30] and more positive implicit responses to social cues [25,31]. Regarding the gene-by-environment framework and the familiar early context, evidence in the literature shows that the 5-HTTLPR polymorphism plays a role in modulating the sensitivity to external factors, enhancing the risk of adverse outcomes, or adaptive magnifying strategies [25]. Moreover, Mileva-Seitz and colleagues found an interaction effect between 5-HTTLPR and perceived early maternal care in response to mothers’ gaze orientation to their infants; in particular, mothers carrying the L-allele variation and recalling a less optimal parental care showed the tendency to orient their gaze away from the infant (more negative response), suggesting that the L-variation confers greater susceptibility to the environmental factors [29]. An investigation on physiological responses to relevant social stimuli found that men with increased sensitivity to external features reporting high levels of perceived maternal overprotection showed a greater heart rate index, showing an opposite pattern compared to the S/S-carriers [32]. Another study found moderating effects of the interplay between 5-HTTLPR and the perception of early caregiving on implicit responses to infant and adults faces, finding that L/L-carriers with a positive experience of parental care displayed implicit positive reactions to infant faces regardless of the class of the stimuli (in-group vs out-group); as for S-carriers, the pattern did not differ significantly in accordance to the diverse stimuli or recalled parental acceptance/rejection [25]. So far, the research typically investigated responses and behavior-oriented to parental practices or related to infant social cues, but little is known concerning social expectation in close adult relationships. Genetic characteristics could play a pivotal role in conferring sensitivity to early caregiving environment and shaping the manifold experiences about social conducts with significant partners [33], which are relatively stable and generally described on two main components, namely anxiety and avoidance [34]. Hence, this research investigates how the different serotonin transporter gene allelic variants and the reminiscence of parental bonding during childhood interact in regulating the experience of close relationships in adulthood.

### 1.3. Aim and Hypothesis

The present research explores the potential influence of the interplay between genetic predispositions and the exposure to an early environment on the social expectations involved in close relationships. For this purpose, the 5-HTTLPR was considered as a genetic variable. The quality of parental bonding in childhood was assessed as a measure of the early environmental context using the Parental Bonding Instrument [8]. Finally, social expectations and attachment status towards the partner were collected by the questionnaire Experience in Close Relationship-Revised [34]. An interaction effect between the 5-HTTLPR polymorphism genotype and the parental bonding scores was hypothesized on the close relationship scores. Given the role of 5-HTTLPR and caregiving behavior in sociability modulation [25,29,32,33], two distinct hypotheses were elaborated for each genetic group. Regardless of sex and age, L-carriers (L/S heterozygous and L/L homozygous) would show secure attachment and positive expectation towards their partner (low levels of anxiety and avoidance) when they reported a positive past of parental caregiving (high levels of parental care or low levels of parental overprotection), but a dysfunctional attachment and negative expectation towards the partner (high levels of anxiety and avoidance) when they recalled an adverse history of parenting (low levels of parental care or high levels of parental overprotection) (Hypothesis 1A). On the other hand, S/S homozygous would exhibit a stable social pattern of the overall anxiety and avoidance from the partner independent of environmental exposure in terms of caregiving experiences (Hypothesis 1B). Based on these hypotheses, expected and unexpected main and genotype-by-environment interaction effects on both adult scores of anxiety and avoidance would be evaluated by univariate and multivariate analyses.

## 2. Methods

### 2.1. Participants

The study was conducted in accordance with the Declaration of Helsinki. The present research is part of a broader project investigating the relationship among genes, psychological traits related to parental bonding and adult relationships, and social behavior in an Italian population [20,25,32,35,36,37,38]. Participants were recruited among undergraduates of the University of Trento (Italy) through public announcements on students’ Facebook groups and rewarded with credits for their participation. Inclusion criteria were not being a parent. The final sample consisted of sixty-five voluntary non-parent adults. The sample was formed by 33 Caucasian males (*M* = 23.85, *SD* = 4.54) and 32 females (*M* = 23.66, *SD* = 3.62).

### 2.2. Data Collection

Two assessments were conducted. Students enrolled in the study provided their email addresses. After informed consent was given, they received the link to the self-report questionnaires, namely (i) *the Parental Bonding Instrument* and (ii) *the Experience in Close Relationship-Revised* which were filled online via Google Modules. Subsequently, participants who fully completed the questionnaires and consented to take part in the genetic assessment received an appointment in the laboratory, where a buccal mucosa sample was collected using a sterile cotton swab and, after 48 h, preserved dried at room temperature in a 2 mL tube for following DNA extraction and genotyping.

### 2.3. Parental Bonding

The Parental Bonding Instrument (PBI) [8] measures the quality of parental caregiving experienced during the first sixteen years of life. Completing this 50-items self-report questionnaire, participants recalled their relationship with their parents and reported their self-representation of past maternal and paternal behavior on a Likert scale from 0 to 3. The PBI (average Cronbach’s α=0.88) offers a consistent insight of functional and dysfunctional parental bonding across the dimensions of maternal care (α=0.92; e.g., “Could make me” “Was overprotective of me”), and paternal care (α=0.94; e.g., “Spoke to me in a warm and friendly voice”) and overprotection (α=0.87; e.g., “Gave me as much freedom as I wanted”) [39,40]. In general, care dimensions positively correlate with positive caregiving: a high parental care score is associated with warm, empathetic, and supporting attachment. Overprotection dimensions negatively correlate with positive caregiving: a high parental overprotection score reveals an oppressive, controlling, and intrusive attachment.

### 2.4. Close Relationship

The Experience in Close Relationship-Revised (ECR-R) [41] assesses the quality of intimate relationship experienced in adulthood. Compiling this 36-item self-report questionnaire, participants evaluated their close relationship with their partner and revealed their expectations towards their lover’s social interaction on a Likert scale from 0 to 7. The ECR-R (average Cronbach’s α=0.91) provides a reliable measure of adult romantic attachment across the two dimensions of anxiety (α=0.91; e.g., “I often worry that my partner will not want to stay with me”) and avoidance (α=0.90; e.g., “I am nervous when partners get too close to me”) [42]. Both anxiety and avoidance subscales are negatively correlated with positive and secure attachment with the partner. A high score in anxiety explains increased possessiveness, the worry of refusal, and fear of abandonment, whereas a high score in avoidance defines an inclination to withdraw from the close relationship and refrain from intimacy and support [34].

### 2.5. Genetic Assessment

The genetic data collection followed the procedure indicated by Bonassi et al. [37]. DNA extraction and genotyping were completed by ACGT, Inc. (Wheeling, IL, USA). From each kit DNA was extracted by the means of Oragene DNA purification reagent and its concentrations were assessed through spectroscopy (NanoDrop Technologies, Wilmington, DE, USA). Each DNA sample was augmented by polymerase chain reaction (PCR) for the SLC6A4 gene 5-HTTLPR region target with the primers 50-CCAGCACCTAACCCCTAAT-30 and 50-AGGGACTGAGCTGGACAACCAC-30 marked with 6-FAM (6-carboxyfluorescein). A PCR reaction of 20 ll composed of 1.5 ll of genomic DNA from the test sample, PCR buffer, 1 mM each of the forward and reverse primers, 10 mM deoxyribonucleotides, KapaTaq polymerase, and 50 mM MgCl${}_{2}$ was administered. Cycling operation provides for 15 min denaturation at 95 ${}^{\circ }$C, and 35 cycles at 94 ${}^{\circ }$C (30 s), 60 ${}^{\circ }$C (60 s), 72 ${}^{\circ }$C (60 s) and a final 10 min step at 72 ${}^{\circ }$C. PCR reactions were genotyped with an ABI 3730xl Genetic Analyzer (Applied Biosystems Inc. Waltham, MA, USA) and equalized with GeneScan 600 LIZ (Applied Biosystems, Inc. Waltham, MA, USA) size standards conducted on each sample. Genotypic data were inspected using GeneMapper ID (Applied Biosystems, Inc. Waltham, MA, USA).

To test the dysfunctional effect of the short allele, participants with at least one L allele (L/L homozygotes or L/S) were categorized into a single L-carriers group, whereas participants with a double short allele were preserved in a unique group (S/S homozygotes). The distribution in our sample was 32% for S/S homozygous and 68% for L-carriers. The frequencies of the genotype were: L/L = 19 (29.23%), L/S = 25 (38.46%), S/S = 21 (31.31%), and the distribution was consistent with the Hardy-Weinberg Equilibrium ($X^{2}$ (1) = 3.44, ns).

### 2.6. Statistical Analysis

Statistical analyses and graphical visualization were carried out in R (R-core base version 4.0.0.). Descriptive statistics of the demographic variables, genotypes, and subscales were analyzed. Questionnaires’ dimensions as continuous variables were examined for normality, whereas skewness [43] and kurtosis [44] values were computed (see Table 1). All the continuous variables displayed skewness and kurtosis values between −1 and 1 and therefore were considered to be normally distributed [45]. Every PBI and ECR-R subscale was also inspected by density, quantile-quantile, and diagnostic plots whose visualization confirmed that the related data had a Gaussian distribution.

One Pearson’s correlation test was preliminary conducted to verify any potential association of age on each ECR-R dimension. One Student’s *t*-test was run to exclude any significant differences of age between the two genetic groups. To control any unexpected effect of sex respectively on each ECR-R dimension, one Welch’s *t*-test and one further Student’s *t*-test was computed for anxiety and avoidance respectively. The same discrete probabilities of frequencies for the two genetic groups divided by sex were ascertained by Pearson’s Chi-squared test with Yates’ continuity correction. At a correlational level, positive and negative associations within and between PBI and ECR-R subscales were also explored [46].

The statistical assumptions for multivariate analysis were successively verified. Given that multivariate normality was already proved, the assumption of homoscedasticity through Bartlett tests of homogeneity of variances in anxiety ($K^{2}$ = 0.08, df = 1, ns) and avoidance ($K^{2}$ = 0.04, df = 1, ns) by genotype was checked. Multicollinearity among predictors (5-HTTLPR and PBI subscales) were inspected for each ECR-R dimension ad dependent variable (DV) by variance inflation factor (VIF) test [47]. As postulated [48], VIF values under 2.5 are recommended and VIF values above 5 generally point to potential issues in evaluating the DV [49]. In this study, all the predictors of ECR-R anxiety and avoidance showed VIF values below 2 (Minimum = 1.022, Maximum = 1.78). Homogeneity of Covariance Matrices for anxiety and avoidance by genotype was also inspected when the same ECR-R subscales were considered as DVs in a multivariate mixed linear model (MMLM) ($x^{2}$ = 0.19, df = 3, ns).

Based on the previous postulations, one MMLM was performed with the two ECR-R dimensions (Anxiety and Avoidance) as the dependent variables, 5-HTTLPR as the unique between-subject factor, and the four PBI dimensions (maternal and paternal care, maternal and paternal overprotection) as continuous predictors (see Formula (1)).

(1) $$ECR_{R}Anxiety,Avoidance=5-\mathrm{HTTLPR}*\left(PBI_{MC}+PBI_{PC}+PBI_{MO}+PBI_{PO}\right)$$

Note that a linear model object applied to a unique MMLM in input in R, returns the results of a separate univariate regression for each DV (here, one for ECR-R Anxiety and one for ECR-R- Avoidance). The variance-covariance matrix of the parameters in the non-linear (e.g., 5-HTTLPR) and linear (e.g., the four PBI subscales) predictors of the model was subsequently computed. The normal distribution of residuals was also validated by the Shapiro-Wilk test (W = 0.99, ns) and skewness computation (0.11).

Given all the validations, one ad-hoc type III Pillai-Bartlett multivariate analysis of covariance (MANCOVA) for multiple regressions [50] was applied to the model of MMLM to further account how ECR-R scores from the two dimensions co-varied as a function of the genotype-by-environment interactions. Type III sum of squares calculation is usually recommended for unbalanced designs or unequal sample size across conditions when testing mixed interactions and main effects (see Formula (1)), as in the fitted model [51]. Pillai’s trace is known to be the most robust method against potential deviations from the MANCOVA’s assumptions than the alternative methods applied to analogous models [52]. A previous sensitivity power analysis in G*Power (power = 0.80) estimated a small effect size equal to 0.13 for a MANCOVA (MANOVA family: special effects and interactions).

Univariate and multivariate main effects of genotype or PBI dimension as well as interactions between genotype and PBI dimension on anxiety and avoidance scores were graphically represented by scatterplots, violin-plots [53] and barplots and further evaluated through post-hoc statistical tests. Addressing concerns of the main effect of genotype, one Student’s *t*-test for each ECR-R subscale was adopted to understand the differences of ECR-R scores between the genetic groups. The degree of association between the PBI scores and the ECR-R scores was tested by Pearson’s *r*. Each significant PBI dimension was also divided into low vs. high groups by the median split procedure. Here, one Welch’s *t*-test on anxiety and one Student’s *t*-test on avoidance measured the differences of ECR-R scores between the PBI groups. For the interaction effects, two Student’s t-tests probed the potential differences between the two genetic carriers within the low vs. high PBI groups on the ECR-R- scores. Fisher’s *z* finally allowed to evaluate the slope divergence between the association of ECR-R and PBI scores for each genetic group.

The magnitude of the significant effects for MMLM, MANCOVA and Student’s *t*-tests were respectively estimated by R squared with confidence intervals [54], eta squared [55] and Cohen’s *d*.

The probability to commit Type I errors determined by multiple tests and Type II errors due to small sample size was overall controlled by the false discovery rate (FDR) correction method of Benjamini-Hochberg [56,57].

## 3. Results

### 3.1. Preliminary Analysis: Age and Sex

In line with the hypothesis, the preliminary analysis did not reveal any significant effect of age and sex on the ECR-R dimensions or genotype. Specifically, participants’ age was positively associated with ECR-R anxiety (*t*(63) = 0.15, *r* = 0.02, ns) and avoidance (*t*(63) = 1.14, *r* = 0.14, ns) with no significant evidence. When the variable sex was considered, no differences between males and females were found on the reported level of anxiety (*t*(55) = −1.23, df = 55, ns, *d*<−0.31) and avoidance (*t*(63) = 1.18, ns, *d*< 0.29). As regards the potential influences on the genotype, age (*t*(63) = 0.27, ns) and sex ($X^{2}$(1) = 0.95, ns) also did not significantly differ between the two groups S/S vs L.

### 3.2. Correlational Analysis: PBI and ECR-R Subscales

**Figure 1:**
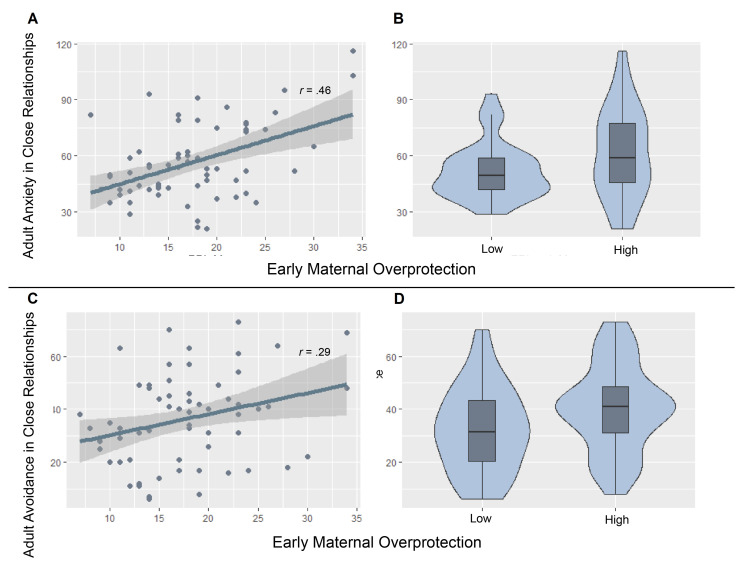
(**A**) Main effect of recalled maternal overprotection in childhood on adult anxiety in close relationships. Pearson *r* coefficients between the early maternal overprotection and anxiety. The line represents the linear model for the total sample. (**B**) Anxiety mean scores by low and high maternal overprotection. (**C**) The main effect of recalled maternal overprotection in childhood on adult avoidance in close relationships. Pearson *r* coefficients between the early maternal overprotection and avoidance. The line represents the linear model for the total sample. (**D**) Avoidance mean scores by low and high maternal overprotection.

Table 2 represents the correlation matrix among PBI and ECR-R subscales. After Benjamini-Hochberg correction, the correlational analysis pointed to a significant positive association between anxiety and avoidance within the ECR-R questionnaire (*t*(63) = 3.73, *r* = 0.43, *p* < 0.01). ECR-R anxiety was also positively associated with PBI maternal overprotection (*t*(63) = 4.13, *r* = 0.46, *p*< 0.001) (see Figure 1A), whereas it was negatively associated with PBI maternal care (*t*(63) = −3.91, *r* = −0.44, *p* = 0.001) and paternal care (*t*(63) = −3.36, *r* = −0.39, *p*< 0.01). ECR-R avoidance was positively correlated with PBI maternal overprotection (*t*(63) = 2.38, *r* = 0.29, *p*< 0.05) (see Figure 1C). As concerns the PBI subscales, a positive correlation between maternal care and paternal care (*t*(63) = 5.78, *r* = 0.59, *p*< 0.0001) as well as between maternal overprotection and paternal overprotection (*t*(63) = 3.08, *r* = 0.36, *p*< 0.01) were detected. A negative correlation was instead found between paternal care and paternal overprotection (*t*(63) = −2.40, *r* = −0.29, *p*< 0.05).

### 3.3. Univariate and Multivariate Analysis: Genotype-by-Environment Interactions on ECR-R Subscales

**Figure 2:**
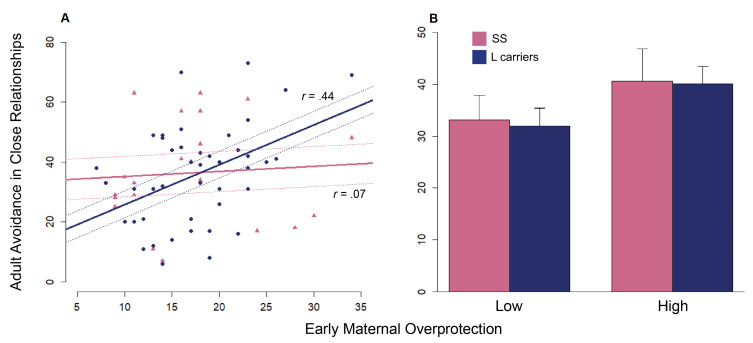
(**A**) Interaction between 5-HTTLPR and maternal overprotection on avoidance. Pearson *r* coefficients between the avoidance scores and the recalled maternal overprotection for each genetic group. Blue circles = L-carriers; pink triangles = S/S. Continuous lines represent the linear models for L-carriers (blue) and S/S homozygous (pink). Dashed lines represent the standard error of the regression lines for L-carriers (blue) and S/S homozygous (pink). (**B**) Avoidance mean scores in L-carriers (blue) and S/S homozygous (pink) by low and high maternal overprotection.

Within the starting MMLM, ECR-R anxiety and avoidance were first treated in separate univariate regressions respectively as DVs. As regards the linear model tested on anxiety (*F*(9, 55) = 3.71, $R^{2}$ (95% CI [0.14, 0.52]) = 0.38), a main effect of early maternal overprotection was discovered (β=1.25, SE = 0.53, *t* = 2.36, *p*= 0.02) (see Table 3 Figure 1A). Results of the linear model performed on avoidance (*F*(9, 55) = 1.93, $R^{2}$ (95% CI [0.03, 0.38]) = 0.38) disclosed a main effect of genotype (β=84.40, SE = 30.52, *t* = 2.77, *p*< 0.008) and early maternal overprotection (β=1.24, SE = 0.48, *t* = 2.58, *p*= 0.01) (see Table 3 Figure 1C). In line with the hypotheses, an interaction effect between genotype and the same PBI covariate (β=−1.87, SE = 0.84, *t* = −2.25, *p*= 0.029) was also observed on the adult avoidance (see Table 4 and Figure 2A,B).

When the two ECR-R subscales were considered in multivariate analysis through the MANCOVA for MMLM, both genotype (Pillai test statistics = 0.13, approximated *F*(2, 54) = 4.12, *p* = 0.02, p$\eta^{2}$ = 0.13) and maternal overprotection (Pillai test statistics = 0.15, *F*(2, 54) = 4.78, *p*= 0.01, p$\eta^{2}$ = 0.15) were found to significantly predict anxiety and avoidance (see Figure 1). However, no interaction effects emerged from the multivariate analysis (see Table 4).

### 3.4. Post-Hoc Analysis: Effects of Genotype and Maternal Overprotection on Avoidance

The direction of the effects from the univariate and multivariate analysis was inspected. As concerns the effect of genotype 5-HTTLPR, anxiety (*t*(63) = −0.50, ns) and avoidance scores (*t*(63) = 0.08, ns) did not differ significantly between S/S homozygous and L-carriers (see Table 3). As regards the effect of maternal overprotection, the difference between participants who reported low and high levels of anxiety (*t*(49) = −1.90, *p*= 0.06) and avoidance (*t*(63) = −1.95, *p*< 0.06) approached the significance (see Table 3 and Figure 1A,B). Furthermore, maternal oveprotection was significantly associated with anxiety and avoidance (see Correlational Analysis and Figure 1A,C).

Limited to the genotype-by-environment interaction detected on avoidance (see Table 4 and Figure 2), maternal overprotection was positively correlated with the avoidance scores for L-carriers (*t*(42) = 3.13, *r* = 0.44, *p* = 0.003). In contrast, no significant association between the same PBI covariate and the avoidance scores for S/S homozygous was found (*t*(19) = 0.30, *r* = 0.07, ns).

## 4. Discussion

This research aimed to probe the potential effect of genetic and early environmental factors on adult social relationships. The interaction between the promoter region of the Serotonin Transporter Gene as a genetic component and the recalled parental caregiving (PBI) as a measure of participants’ family environment during childhood was hypothesized to regulate the adult social expectations on close relationships (ECR-R). Therefore, one gene*environment interaction (5-HTTLPR polymorphism*parental bonding in childhood) was predicted on the two ECR-R dimensions anxiety and avoidance in an Italian sample. Participants’ age and sex were also not expected to moderate the gene*environment interaction on the ECR-R dependent variables.

The preliminary analysis confirmed that participants’ age and sex did not significantly moderate genotype and adult anxiety and avoidance. The results highlighted several significant associations within and between each questionnaire once the correction for multiple tests was applied. Adult anxiety was positively correlated with adult avoidance and maternal overprotection in childhood but negatively correlated with early maternal and paternal care. Adult avoidance was instead positively associated with maternal overprotection. A positive relationship between maternal care and paternal care as well as between maternal and paternal overprotection was established. In contrast, paternal care was inversely related to paternal overprotection. The multivariate analysis revealed that genotype and maternal overprotection in childhood were positive predictors of anxiety and avoidance in adulthood. Maternal overprotection was also found to explain a significant portion of the variance in anxiety and avoidance when the ECR-R variables were considered in the univariate analysis. The interaction between genotype and recalled maternal overprotection affected the reported levels of adult avoidance in line with the assumptions. Although the post-hoc analysis did not detect significant mean differences between the genetic groups, as expected, the positive association between avoidance and maternal overprotection was significant for L-carriers but not for S/S homozygous.

The present study extends previous knowledge on parent-infant attachment and adult attachment which characterizes social development [58,59,60]. The observed correlations between PBI and ECR-R dimensions almost replicate the attachment patterns reported by previous studies on an Italian sample [20]. These results indicate that the reported scores in parental caregiving are associated with the levels of adult attachment at distinct directionality: the higher the scores in parental (e.g., maternal) overprotection dimension, the higher the scores in anxiety and avoidance dimensions; conversely, the higher the scores in parental care dimensions, the lower the scores in the same ECR-R dimensions. Not only, recalled maternal overprotection in childhood predicted the anxiety and avoidance felt towards the partner in adulthood [61]. A recent work describes that a more severe grief consequent to the romantic breakup was linked to child maltreatment [62]. The evidence of a linear relationship between the PBI maternal overprotection and the ECR-R subscales meets the starting assumption on the prototype hypothesis, which postulates that the early relationship with the parents represent a shared basis for subsequent social experiences [63], underpinning romantic functioning and believes [64]. In reference to the attachment theory [65], individuals who experienced a negative relationship with the parent (high scores in maternal overprotection) would have shown higher levels in anxiety and avoidance in romantic relationship [20], as witnessed by the heightened scores obtained in the ECR-R dimensions. The current data suggest that a history of adverse maternal bonding may have a long-term impact on the adult attachment status [66]. Specifically, the presence of an overprotective mother may be pervasive and characterized by high control of child’s life, interference with child’s decision, tendency to treat the child in a babyish manner. The child’s dependence on the mother, in turn, could induce perspective emotional feelings of anxiety in intimate relationships. An anxious individual may exhibit an insecure attachment with the partner, being afraid that his/her love may not be reciprocated or that he/she could be neglected or abandoned by the lover. Similarly, a maternal suppression of autonomy in childhood could exacerbate the sense of restriction perceived in the couple. High avoidance from the partner echoes an adverse history of maternal overprotection. However, the distinct slopes of the lines depicting the two genetic groups showed a differential avoidant response when exposed to a familiar environment of dysfunctional maternal bonding. In accordance with the hypotheses, individuals with the genotype sensitive to the environment (L-carriers) seemed more affected by a past of maternal overprotection than people genetically less sensitive to early conditions of social distress (S/S homozygotes). This finding agrees with previous literature, which identified L-carriers as more susceptible to environmental influences than S/S homozygotes as theorized by the sensitivity hypothesis of genes [32]. No effect was found for the interaction between recalled parental bonding and 5-HTTLPR on anxiety experienced in adult relationships, suggesting that mechanisms related to preoccupation are less or not regulated by this polymorphism, while the perception of maternal features, predominantly the overprotection, explain better the concerns with close bonds. Similarly, in their developmental analysis on the genetic modulation of early care and social functioning, Belsky and Pluess did not find any significant result for 5-HTTLPR, indicating that, although surprising, the result was consistent with the differential susceptibility theory [67]. Moreover, while avoidance clearly describes withdrawal as form of distress, anxiety in close relationships could be recognized as a form of excessive care, especially in a country like Italy where higher levels of emotions are expressed in significant bonds, thus resulting in more associated with parental characteristics rather than genetic ones [20].

Since a significant mean difference of reported avoidance between the genetic groups divided by low and high maternal overprotection was not ascertained, cautiousness should be exercised to interpret the data. Further studies will be required to understand whether the short or long allele would confer a higher attitude to avoid the partner when undermined by harmful maternal caregiving (i.e., high maternal overprotection). For instance, the short allele of 5-HTTLPR was associated with unresolved attachment in a sample of adoptees with predominant Caucasian ethnicity [68]. A previous study suggested that Italian males with the long allele and a past of maternal overprotection show higher physiological distress to woman cry than S/S homozygous, but the opposite scenario was confirmed in a context of favorable parenting [32]. Some contrasting evidence points to the absence of association between 5-HTTLPR and adult attachment [69], thus leading to carefully interpreting the potential effect of parent-infant attachment on close relationship independent of the genetic profile. A piece of research recently discovered on a Singaporean sample that another region of the Serotonin Transporter Gene (i.e., 5HTT rs25531) is associated with parental overprotection in modulating anxiety levels towards the partner independent of age and sex [70].

Overall, this study substantiates that the quality of parental bonding in childhood may affect the expectations on close relationships in adulthood [71,72,73]. In support of meaningful researches, this work proposes that target genetic predispositions (e.g., 5-HTTLPR) and the quality of familiar environment (e.g., parental bonding) may represent postnatal longitudinal predictors of typical and atypical social development [74].

## 5. Conclusions and Limitations

In this study, we framed the romantic attachment from a gene*environment perspective. Intending to further shed light on the potential involvement of early life stressors on adult social life, we investigated how Serotonin Transporter Gene combined with the recalled parental bonding regulate adult expectations on close relationships. The opportunity to scrutinize the main and interaction effects of genetic predispositions and the early environment through conservative univariate and multivariate analysis offers a comprehensive view of adult attachment. Interestingly, we observed that the effect of parental caregiving on the close relationship was mainly driven by maternal overprotection, underlining the centrality of maternal involvement in child development. This work advances a further step in the research field of developmental psychobiology and could be of clinical relevance for professionals who operate in the field of mental health. In particular, the current results could support the understanding of the intertwined social dynamics throughout development. The observed link between the perception of the relationship with parents during childhood and the expectation on adult attachment could disclose shared mechanisms behind these different forms of social interactions, from the early experiences with the parents to the adult relationships with the partner. For instance, the transmission of attachment and affection with the caregiver across generations has been witnessed by previous investigations [75,76], highlighting how the detrimental consequence of traumatic events or experiences (e.g., a history of maternal overprotection) could worsen developmental outcomes in individuals sensitive to adversities. The same adult attachment can predict the security of the subsequent child attachment with the parents, revealing that appropriate parental responsiveness to child signals may have a crucial role in regulating the socio-emotional functioning of child (and future adult) behavior [77,78].

This study also presented some limitations which should be addressed in future researches. Firstly, study constraints bounded the sample size. Our targeted pool of participants, together with our inclusion criteria, limited the overall number of participants we were able to recruit. Candidate gene association research generally requires a greater number of participants to increase the statistical power and decrease the probability of a Type II error, strengthening the results. However, a more conservative approach and meticulous statistical procedure were applied to reduce the likelihood of committing Type I errors. A sensitivity analysis, conducted in G*Power, revealed that with a compromise on the power of the test (power = 0.80), our analysis is able to detect small effect sizes (0.13). We are planning for future works to collect genetic data from a larger sample, in order to replicate our findings with a higher statistical power. Additionally, future works should take into account the functionality of the single nucleotide polymorphism rs25531. In fact, the G allele of rs25531 renders the 5-HTTLPR L allele functionally equivalent to the S allele. Despite that, previous work that investigated both 5-HTTLPR and rs25531 SNP reported that the incidence of this particular combination in a similar sample to the one here tested to be lower than 10% of the population [79]. For these reasons, we believe that extending the analysis to the rs25531 would produce similar results to those presented in this work. Moreover, the PBI and the ECR-R are self-reported questionnaires whose answers depend on participants’ responses. A longitudinal approach with follow-up could instead guarantee an objective observation of the different stages of social development from infancy to adulthood. The socio-demographic investigation was limited to age, sex, and the current relational status, and the presence of mental health issues or psychiatric disorders was not evaluated, representing a further limitation to the study, as levels of avoidance and anxiety in relationships could be caused by psychological distress or psychopathology. Moreover, early life traumatic events involving the parent-child relationship (e.g., separation from parent, bereavement) were not controlled and could have affected responses to the questionnaires. However, only fully completed surveys, including both maternal and parental forms of the PBI were considered in the study.

Further contributions could confirm the association between 5-HTTLPR and adult attachment measured by distinct interviewer-assessed (e.g., Current Relationship Interview, Couple Attachment Interview, Secure Base Scoring System) and self-reported instruments (e.g., Adult Attachment Styles, Adult Attachment Questionnaire, Revised Adult Attachment Questionnaire, Attachment History Questionnaire, Relationship Scales Questionnaire) [80]. At a genetic level, the moderating role of the oxytocin receptor gene (OXTR) and dopamine receptor gene (DRD4) on adult attachment could be further explored. Here, only the Caucasian ethnicity has been examined. Given the substantial influence that culture and society have on human development [20], subsequent works could inspect significant gene-by-environment interactions across different cultures and countries. As the recalled parental behavior have been proved to affect the transgenerational transmission of rearing practices and the quality of the bond with the child [81,82], future studies should adopt a gene-by-environment approach to investigate the impact of parenthood and transition to parenting in new parents and on the relationship with the newborn.

## Figures and Tables

**Table 1:** Descriptive statistics for the distribution of each questionnaire’s subscale: Minimum (Min), Median, Mean, Maximum (Max), Standard Deviation (SD), Skewness and Kurtosis. Top section: PBI dimensions. Bottom section: ECR-R dimensions.

Dimension	Min	Median	Mean	Max	SD	Skewness	Kurtosis
Maternal Care	4.00	27.00	25.97	36.00	8.33	−0.95	0.11
Maternal Overprotection	7.00	17.00	17.45	34.00	6.00	0.66	0.17
Paternal Care	1.00	25.00	22.91	36.00	9.44	−0.51	−0.63
Paternal Overprotection	3.00	15.00	15.51	33.00	7.36	0.66	−0.19
Anxiety	21.00	53.00	56.40	116.00	20.19	0.72	0.02
Avoidance	6.00	38.00	36.14	46.00	73.00	0.18	−0.65

**Table 2:** Pearson correlation coefficients and significance levels among questionnaires’ subscales (PBI: Maternal Care, Maternal Overprotection, Paternal Care, Paternal Overprotection; ECR-R: Anxiety, Avoidance). Significance is corrected for multiple tests. * *p* < 0.05, ** *p* < 0.01, *** *p* < 0.001, **** *p* < 0.0001.

*Subscale*	Maternal Care	Maternal Overp	Paternal Care	Paternal Overp	Anxiety	Avoidance
**Maternal Care**		−0.26	0.59 ****	0.02	−0.44 ***	−0.22
**Maternal Overp**			−0.23	0.36 **	0.46 ***	0.29 *
**Paternal Care**				−0.29 *	−0.39 **	−0.23
**Paternal Overp**					0.14	0.08
**Anxiety**						0.43 **
**Avoidance**						

**Table 3:** Mean values of variables with main effects. Top section: mean values in 5-HTTLPR S/S homozygotes and L-carriers on the ECR-R variables. Below section: mean values in low and high maternal overprotection on the ECR-R variables. Standard error means are reported between parentheses.

ECR-R dimension	L	S/S
Anxiety	57.27 (3.01)	54.57 (4.59)
Avoidance	36.02 (2.49)	36.38 (3.75)
**ECR-R Dimension**	**Low Maternal Overprotection**	**High Maternal Overprotection**
Anxiety	51.85 (2.56)	61.39 (4.31)
Avoidance	32.38 (2.76)	40.26 (2.95)

**Table 4:** Mean values in 5-HTTLPR L-carriers and S/S homozygotes by low and high PBI dimensions on ECR-R variables. Standard error means are reported between parentheses.

ECR-R Anxiety				
**PBI Dimension**	**Low/L**	**Low/SS**	**High/L**	**High/SS**
Maternal Care	65.00 (4.47)	62.73 (6.98)	49.55 (3.37)	45.60 (4.68)
Maternal Overprotection	53.14 (3.32)	49.50 (4.06)	61.41 (4.94)	61.33 (9.10)
Paternal Care	64.38 (4.16)	59.23 (6.74)	48.75 (3.57)	47.00 (4.32)
Paternal Overprotection	55.00 (3.89)	53.36 (7.79)	59.55 (4.63)	55.90 (4.89)
**ECR-R Avoidance**				
**PBI Dimension**	**Low/L**	**Low/SS**	**High/L**	**High/SS**
Maternal Care	41.14 (3.59)	42.46 (4.99)	30.91 (3.17)	29.07 (5.06)
Maternal Overprotection	31.96 (3.50)	33.17 (4.67)	40.09 (3.41)	40.67 (6.16)
Paternal Care	38.33 (3.76)	39.77 (4.83)	33.25 (3.10)	30.88 (5.76)
Paternal Overprotection	36.14 (3.14)	38.00 (5.66)	35.91 (3.94)	34.60 (5.06)

## Data Availability

The data of this study can be found in the Nanyang Technological University’s Data repository (DR-NTU Data) at the following address: https://doi.org/10.21979/N9/SZNMRI (accessed on 5 August 2021).

## Abbreviations

The following abbreviations are used in this manuscript:

5-HTTLPR	Serotonin Transporter linked polymorphic region
ECR-R	Experience in Close Relationship-Revised
MMLM	Multivariate Mixed Linear Model
PBI	Parental Bonding Instrument
